# Progressive iron overload in middle-aged mice impairs olfactory function, triggers lipid oxidation and induces apoptosis

**DOI:** 10.3389/fphar.2024.1506944

**Published:** 2024-12-19

**Authors:** Lin Deng, Qihui Luo, Yucong Liu, Yao Wang, Zongliang Xiong, Hongping Wang, Lu Zhao, Lanlan Jia, Riyi Shi, Chao Huang, Zhengli Chen

**Affiliations:** ^1^ Laboratory of Experimental Animal Disease Model, College of Veterinary Medicine, Sichuan Agricultural University, Chengdu, China; ^2^ Key Laboratory of Animal Disease and Human Health of Sichuan Province, College of Veterinary Medicine, Chengdu, China; ^3^ Safety Evaluation Center, Sichuan Institute for Drug Control (Sichuan Testing Center of Medical Devices), Chengdu, China; ^4^ Department of Basic Medical Sciences, Center for Paralysis Research, College of Veterinary Medicine, Purdue University, West Lafayette, IN, United States

**Keywords:** iron overload, mice, lipid peroxidation, olfactory, apoptosis

## Abstract

**Introduction:**

This study aims to investigate the progressive impact of chronic iron overload on the olfactory bulb, a region significantly affected in early neurodegenerative diseases like Parkinson’s and Alzheimer’s. The focus is on understanding how iron accumulation leads to oxidative stress, mitochondrial dysfunction, and neuronal damage over time in middle-aged mice.

**Method:**

The mice were continuously administered FC for a duration of 16 weeks, and the olfactory behavior of the mice was observed at intervals of 4 weeks. Inductively coupled plasma mass spectrometry (ICP-MS) was employed to detect alterations in iron content within the olfactory bulb of the mice, while levels of lipid peroxidation and antioxidant indexes were assessed using biochemical kits. Additionally, western blotting and qPCR techniques were utilized to analyze transcriptional and expression changes in proteins and genes related to iron metabolism. Furthermore, microstructural modifications as well as mitochondrial observations were conducted through paraffin sectioning and transmission electron microscopy (TEM).

**Result:**

A significant and progressive increase in iron accumulation in the olfactory bulb, starting from week 8 and peaking at week 16. This accumulation coincided with a decline in olfactory function observed at week 12. Key markers of oxidative stress, such as 4-HNE and MDA, were elevated in specific layers, and antioxidant defenses were reduced. Mitochondrial damage became evident from week 8, with caspase-3 activation indicating increased apoptosis, particularly in the granular layer. This study is to demonstrate the link between chronic iron overload and progressive olfactory dysfunction in the context of neurodegenerative diseases. It provides evidence that iron-induced oxidative stress and mitochondrial damage in the olfactory bulb contribute to early sensory deficits, suggesting that the olfactory bulb’s selective vulnerability can serve as an early biomarker for neurodegenerative conditions.

**Conclusion:**

Chronic iron overload leads to progressive oxidative damage, mitochondrial dysfunction, and apoptosis in the olfactory bulb, causing sensory deficits. Targeting iron accumulation and oxidative damage may offer new strategies for early intervention in neurodegenerative diseases, highlighting the importance of addressing iron dysregulation.

## 1 Introduction

The olfactory bulb, a critical brain region involved in the initial processing of olfactory information, plays a key role in diverse functions, from odor detection to memory formation and emotional regulation. In neurodegenerative diseases such as Parkinson’s and Alzheimer’s, damage to the olfactory bulb often represents one of the earliest clinical manifestations, frequently preceding the onset of more overt motor or cognitive symptoms (Jellinger, 2024; Park and Schott, 2022; Rey et al., 2018). This early manifestation of olfactory deficits suggests that the olfactory bulb may be among the first regions affected during the initial stages of neurodegenerative processes. However, the precise molecular and cellular mechanisms underlying the loss of olfactory function in these diseases remain poorly understood (Marin et al., 2018).

Iron, an essential trace element, has emerged as a crucial factor in the pathogenesis of various neurodegenerative diseases (Jellinger, 2013; Mezzaroba et al., 2019). While iron is vital for numerous physiological processes, including oxygen transport, mitochondrial respiration, and neurotransmitter synthesis, its ability to catalyze the formation of reactive oxygen species (ROS) through the Fenton reaction can result in oxidative stress, mitochondrial dysfunction, and neuronal damage—key processes implicated in neurodegeneration. Existing research has demonstrated that iron dysregulation contributes to neurodegenerative processes, with evidence of iron accumulation in specific brain regions, such as the substantia nigra in Parkinson’s disease (Du et al., 2022; Li et al., 2022; Mochizuki et al., 2020) and the hippocampus in Alzheimer’s disease (Cirovic et al., 2023), where it is associated with motor and cognitive decline (Spence et al., 2020). In these regions, iron accumulation promotes neurotoxicity through pathways involving oxidative damage (Hinarejos et al., 2020), protein aggregation (D’Mello and Kindy, 2020), and cellular apoptosis (Wang et al., 2024). Clinical observations suggest that olfactory dysfunction often precedes motor symptoms in neurodegenerative diseases (Fatuzzo et al., 2023), indicating that the olfactory bulb could potentially serve as an early marker for systemic iron dysregulation. Although iron accumulation in specific brain regions is linked to conditions like Parkinson’s (Li et al., 2022), its impact on the olfactory bulb—a region with dense dopaminergic networks and high metabolic activity—remains largely unexplored. Investigating how iron dysregulation affects the olfactory bulb could provide crucial insights into early pathophysiological changes, potentially offering a biomarker for earlier diagnosis and intervention. Additionally, the potential for chronic iron supplementation to exacerbate brain iron accumulation and oxidative stress underscores the importance of understanding iron overload’s toxic effects on olfactory function and neuronal integrity.

To address key gaps in understanding iron-induced neurotoxicity, this study investigates the progressive effects of chronic ferric citrate supplementation on the olfactory bulb of middle-aged mice over 16 weeks. We employed advanced imaging and biochemical techniques, including inductively coupled plasma mass spectrometry (ICP-MS) and Perls’ Prussian blue staining, to quantify and localize iron accumulation at 4, 8, 12, and 16 weeks. Oxidative stress markers such as 4-hydroxynonenal (4-HNE) and malondialdehyde (MDA) were measured to assess lipid peroxidation and neuronal damage, while caspase-3 activation was tracked to elucidate the progression of iron-induced apoptosis. Mitochondrial changes were evaluated using transmission electron microscopy (TEM) to identify structural alterations due to oxidative stress. This comprehensive temporal analysis reveals the mechanisms underlying the olfactory bulb’s heightened vulnerability to iron overload and supports its potential role as an early biomarker for neurodegenerative diseases, offering insights that may guide the development of early interventions to prevent neuronal damage.

## 2 Results

### 2.1 Selective iron accumulation in the olfactory bulb leads to sensory dysfunction in mice

Disruption of iron homeostasis is increasingly recognized as a pivotal factor in the pathogenesis of neurodegenerative diseases, particularly in brain regions characterized by high metabolic activity and dense dopaminergic networks, such as the olfactory bulb (Wise et al., 2022). These regions are highly susceptible to iron-induced oxidative stress, which promotes the generation of reactive oxygen species (ROS), leading to cellular damage through lipid peroxidation, protein misfolding, and DNA damage (Merelli et al., 2021; Umare et al., 2022). Recent studies underscore the critical role of iron dysregulation in the early sensory deficits often associated with neurodegenerative disorders like Parkinson’s and Alzheimer’s diseases (David et al., 2022; Pal et al., 2022).

**FIGURE 1:**
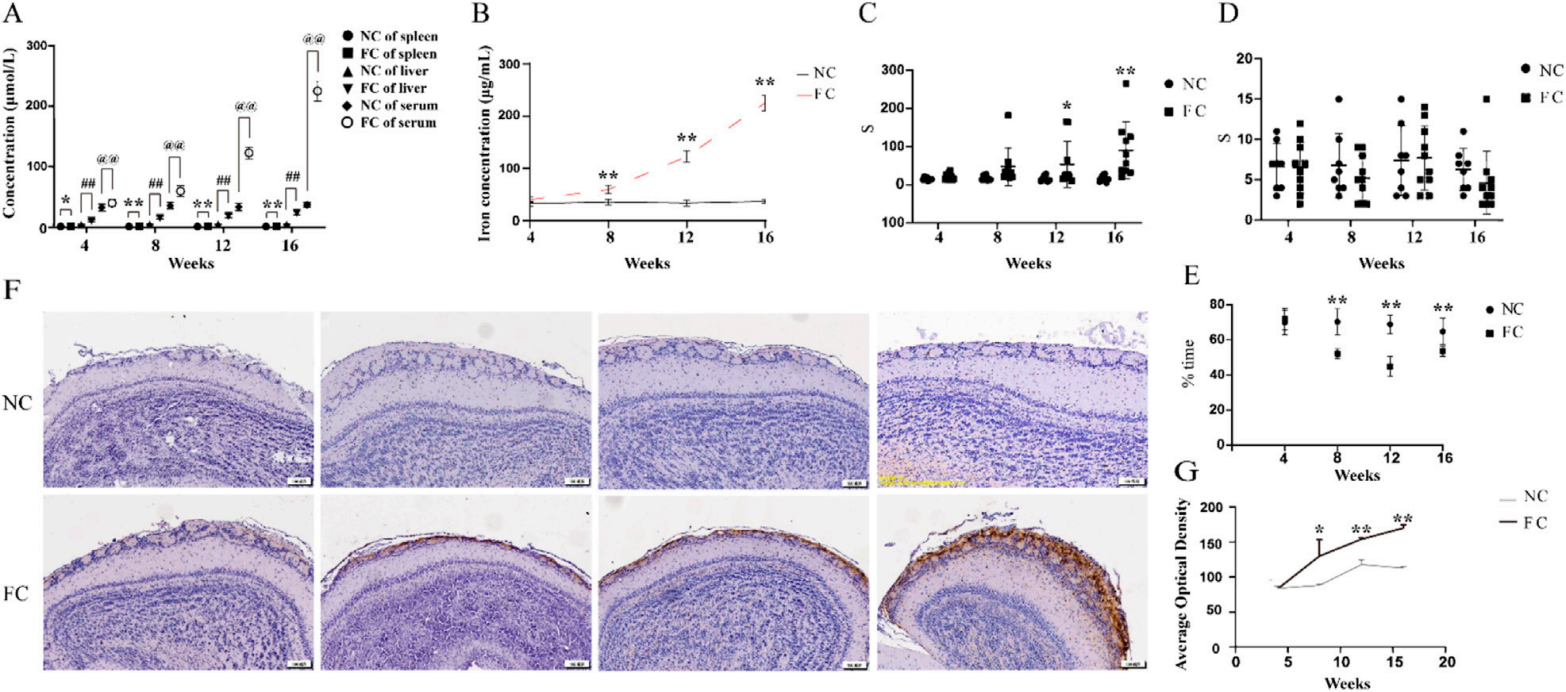
Selective iron accumulation in the olfactory bulb and impaired olfactory function following chronic iron overload in middle-aged mice **(A)**. Quantification of iron concentration in the serum, spleen, and liver of ferric citrate-treated (FC) and normal control (NC) mice over time, measured by inductively coupled plasma mass spectrometry (ICP-MS). In the FC group, a significant increase in iron concentration was detected in the serum from week 4, with further elevations at weeks 8, 12 and 16 (@@*P* < 0.01), while no significant changes were observed in the NC group. Similarly, the spleen and liver of FC mice showed marked iron accumulation starting at week 4 (spleen, *P < 0.05; liver, ##*P* < 0.01) and continuing to rise through weeks 8, 12 and 16 (**P < 0.01, ##*P* < 0.01), with no corresponding increase in the NC group. **(B)** Quantification of iron concentration in the olfactory bulb over time in NC and FC mice. From week 8 onwards, there is a marked exponential increase in serum ferric in the FC group, significantly higher than the control group (**P < 0.01). The control group maintains stable iron levels across all time points. **(C)** Latency to find buried food in the food-burial test as a measure of olfactory function in NC and FC mice over time. FC-treated mice exhibited a significant increase in the time required to find buried food compared to the NC group, starting at week 12 (*P < 0.05) and becoming more pronounced at week 16 (**P < 0.01), indicating impaired olfactory function with increasing iron accumulation. **(D)** Latency to find food on the surface in the surface food-finding test as a measure of olfactory function in NC and FC mice over time. No significant difference was observed in the time required to find food placed on the surface between FC-treated and NC mice across all time points, suggesting that superficial olfactory detection ability remains unaffected despite iron overload. **(E)** The ratio of time remained in familiar to unfamiliar (Familiar/Unfamiliar) space of NC and FC mice. From 8 to 16 weeks, the ratio declined significantly in FC mice compared with NC (**P < 0.01). **(F)** Representative histological sections of the olfactory bulb from NC and FC mice at 4, 8, 12, and 16 weeks, stained with Perls’ Prussian blue to detect ferric iron deposition. FC-treated mice show progressively increased ferric staining from week 8 onward, while NC mice display consistently minimal staining, indicating localized iron accumulation in the olfactory bulb over time. **(G)** Quantitative analysis of ferric-positive areas in the olfactory bulb, corresponding to panel **(F)** A significant increase in ferric deposition is observed in the FC group starting at week 8, with further escalation by week 16 (**P < 0.01), while the NC group shows no substantial change, corroborating the progressive accumulation of iron in the olfactory bulb.

Our study provides new insights into the consequences of chronic iron overload on sensory function, specifically highlighting the selective vulnerability of the olfactory bulb in this context. We observed a significant and progressive elevation in serum iron levels in ferric citrate (FC)-treated mice starting from week 8, coupled with a marked accumulation of iron in the olfactory bulb that peaked by week 16 (Figures 1A, B, F). This accumulation was visually confirmed by intensified Perls’ Prussian blue staining, which revealed increasing ferric deposition over time (Figure 1F), and quantitatively supported by a substantial rise in ferric-positive areas in the olfactory bulb (Figure 1G). Unlike the olfactory bulb, other organs, including the liver and spleen, exhibited different patterns of iron accumulation, underscoring the unique vulnerability of the olfactory bulb to iron overload (Figure 1B).

The selective iron deposition in the olfactory bulb is likely driven by its distinct physiological and metabolic characteristics. The olfactory bulb’s high demand for oxygen and nutrients, coupled with its rich dopaminergic innervation and active iron metabolism, creates a local environment that is particularly prone to iron accumulation and oxidative stress. This environment sets the stage for iron-induced neurotoxicity, where excess iron catalyzes the formation of ROS, exacerbating oxidative damage and impairing cellular function. This is reflected in our findings, where FC-treated mice showed a significant decline in olfactory performance, evidenced by increased latency to locate buried food from week 12 onward (Figure 1C), while the latency to find food on the surface remained unaffected (Figure 1D). Meanwhile, the same trend was similarly observed in the olfactory discrimination test, The FC group ratio (Familiar/Unfamiliar) experienced a significant decrease (Figure 1E). This selective behavioral deficit indicates that the deeper olfactory processing required for detecting buried food is particularly susceptible to iron-induced dysfunction. The progressive nature of iron accumulation in the olfactory bulb and its correlation with the decline in olfactory function in FC-treated mice align with the notion that sensory deficits may serve as early indicators of iron-mediated neurodegenerative processes. The olfactory bulb, given its unique anatomical and functional attributes, may act as an early sentinel of systemic iron dysregulation, where even moderate increases in iron can trigger a cascade of events leading to sensory impairment. This is consistent with clinical observations that olfactory impairments often precede motor symptoms in conditions like Parkinson’s and Alzheimer’s diseases (Dan et al., 2021; Fatuzzo et al., 2023), suggesting that the olfactory bulb may be among the first brain regions to exhibit dysfunction due to iron overload.

### 2.2 Compensatory upregulation of iron metabolism and lipid oxidation pathways in response to iron overload in the olfactory bulb

**FIGURE 2:**
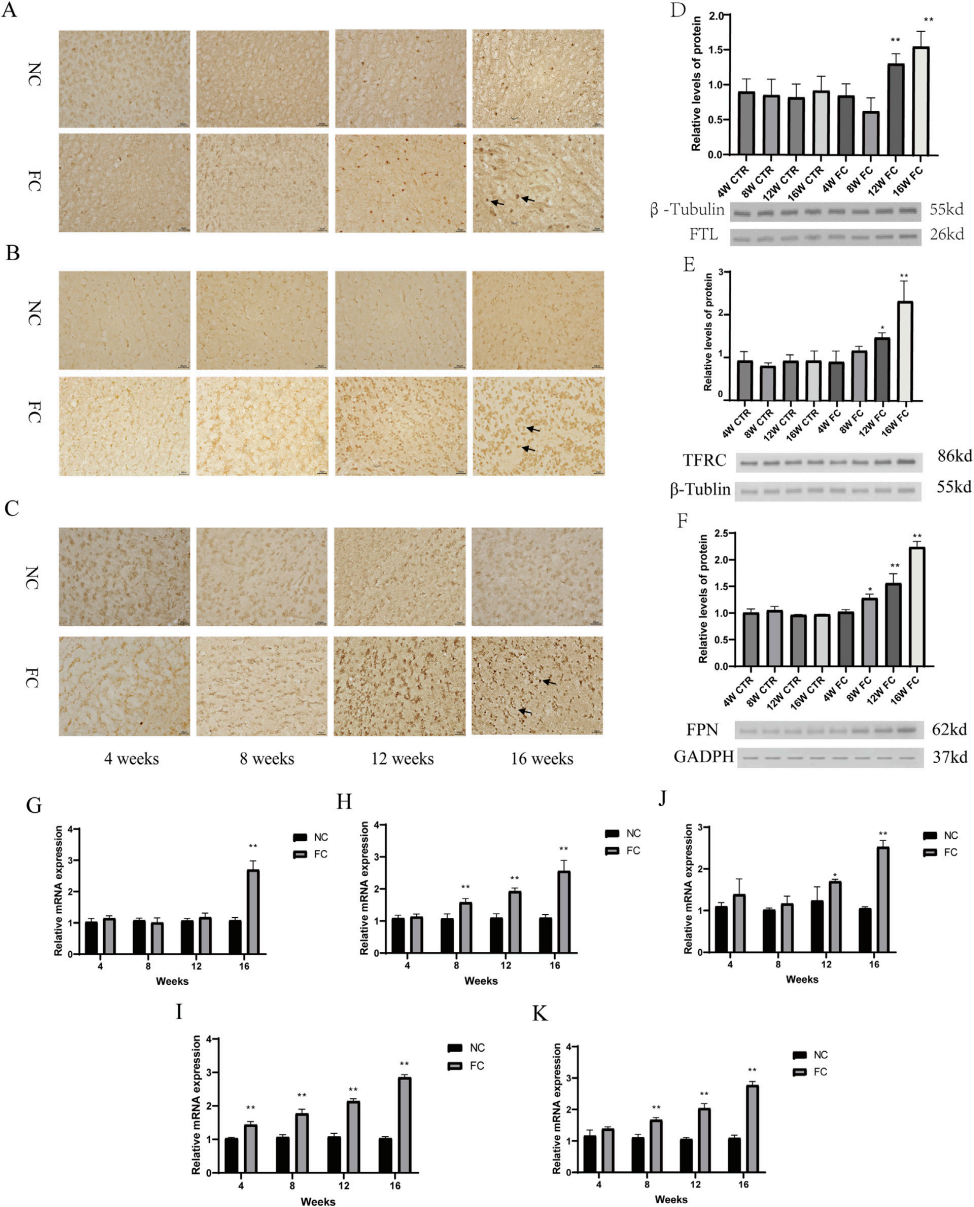
Compensatory upregulation of iron metabolism and lipid oxidation pathways in response to iron overload in the olfactory bulb. **(A–C)** Immunohistochemical analysis of ferritin light chain FTL, **(A)**, transferrin receptor TFRC, **(B)** and ferroportin FPN, **(C)** in the olfactory bulb of NC (normal control) and FC (ferric citrate-treated) mice at 4, 8, 12, and 16 weeks. FC-treated mice show progressively increased expression of FTL, TFRC, and FPN over time, with more pronounced staining starting at week 8 and becoming markedly evident by week 16, suggesting a compensatory upregulation in response to iron overload. **(D–F)** Western blot analysis of FTL **(D)**, TFRC **(E)** and FPN **(F)** in the olfactory bulb of NC and FC mice at 4, 8, 12, and 16 weeks. In FC-treated mice, FTL levels are significantly elevated at weeks 12 and 16 (**P < 0.01; **(D)**), TFRC levels increase significantly at week 16 (*P < 0.05) and are highly significant (**P < 0.01) at week 16 **(E)**, and FPN levels are significantly higher at week 12 (*P < 0.05) and week 16 (**P < 0.01; **(F)**) compared to the NC group. **(G–K)** Quantitative PCR (qPCR) analysis of mRNA expression for FTL **(G)**, TFRC **(H)**, FPN **(I)**, SCL7A11 **(J)** and ACSL4 **(K)** in the olfactory bulb of NC and FC mice at 4, 8, 12, and 16 weeks. The FC group shows a significant increase in FTL mRNA at week 16 (**P < 0.01; **(G)**), in TFRC mRNA levels from week 8 (**P < 0.01; **(H)**), in FPN mRNA levels at week 12 (*P < 0.05) and week 16 (**P < 0.01; **(I)**), in SCL7A11 mRNA levels from week 4 to week 16 (**P < 0.01 for all time points; **(J)**), and in ACSL4 mRNA levels from week 4 onward (**P < 0.01; **(K)**), indicating a robust and coordinated transcriptional response to iron overload and associated oxidative stress.

Iron dysregulation has emerged as a critical factor in neurodegenerative diseases, contributing to neuronal damage through oxidative stress and altered metabolic pathways (Klemmensen et al., 2023; Rehman et al., 2023). While much research has focused on regions such as the substantia nigra (Liu et al., 2023) and hippocampus (Levi et al., 2024), the impact of iron overload on the olfactory bulb remains less explored. In our study, we observed a significant compensatory upregulation of iron metabolism proteins in response to chronic iron overload in the olfactory bulb. Immunohistochemical analysis demonstrated progressively increased expression of ferritin light chain (FTL), transferrin receptor (TFRC), and ferroportin (FPN) starting at week 8 and becoming markedly evident by week 16 in FC-treated mice (Figures 2A–C). This was further confirmed by Western blot analysis, which showed significantly elevated levels of FTL at weeks 12 and 16 (**P < 0.01; Figure 2D), with TFRC and FPN levels also significantly increased at week 16 (*P < 0.05 and **P < 0.01; Figures 2E, F). These results suggest an adaptive response to manage excess iron by enhancing iron storage and export mechanisms.

In parallel with these changes in iron metabolism, our findings highlight a robust activation of oxidative stress and lipid oxidation pathways. Quantitative PCR analysis revealed a significant upregulation of FTL and TFRC mRNA levels from week 8 onwards (**P < 0.01; Figures 2G, H), and FPN mRNA levels at weeks 12 and 16 (*P < 0.05, **P < 0.01; Figure 2I), reflecting ongoing iron-handling efforts. Notably, there was also a marked increase in the expression of SCL7A11, a key component of the cellular antioxidant defense system, from week 4 to week 16 (**P < 0.01 for all time points; Figure 2J). Additionally, the significant elevation of ACSL4 mRNA levels from week 4 onward (**P < 0.01; Figure 2K) indicates that lipid peroxidation pathways are also activated early in response to iron-induced oxidative stress. This coordinated response reflects the olfactory bulb’s attempt to mitigate damage through both iron sequestration and the management of lipid oxidation products. The simultaneous upregulation of iron metabolism and lipid oxidation markers suggests that the olfactory bulb engages multiple defensive strategies to cope with iron overload and its associated oxidative damage. However, the persistence of these responses over time indicates that the compensatory mechanisms may eventually be overwhelmed, potentially leading to further cellular dysfunction. The early and sustained increases in SCL7A11 and ACSL4 highlight the dual challenge of managing both iron homeostasis and lipid peroxidation, suggesting that the olfactory bulb may serve as a critical site for early neurodegenerative changes where both iron dysregulation and lipid oxidation play pivotal roles.

### 2.3 Progressive neuronal and mitochondrial dysfunction in the olfactory bulb induced by chronic iron overload

Iron dysregulation is a critical factor in the development of neurodegenerative diseases, with growing evidence showing that chronic iron overload drives progressive neuronal damage through oxidative stress and inflammation. Although iron is essential for functions such as oxygen transport, DNA synthesis, and mitochondrial electron transport, excess iron catalyzes the formation of reactive oxygen species (ROS), leading to oxidative damage of proteins, lipids, and DNA (Jomova et al., 2023; Juan et al., 2021). Mitochondria, central to cellular energy production, are particularly vulnerable to iron-induced oxidative stress, making mitochondrial dysfunction a key contributor to neuronal degeneration in disorders like Alzheimer’s and Parkinson’s diseases (Cheng et al., 2021; Wise et al., 2022).

**FIGURE 3:**
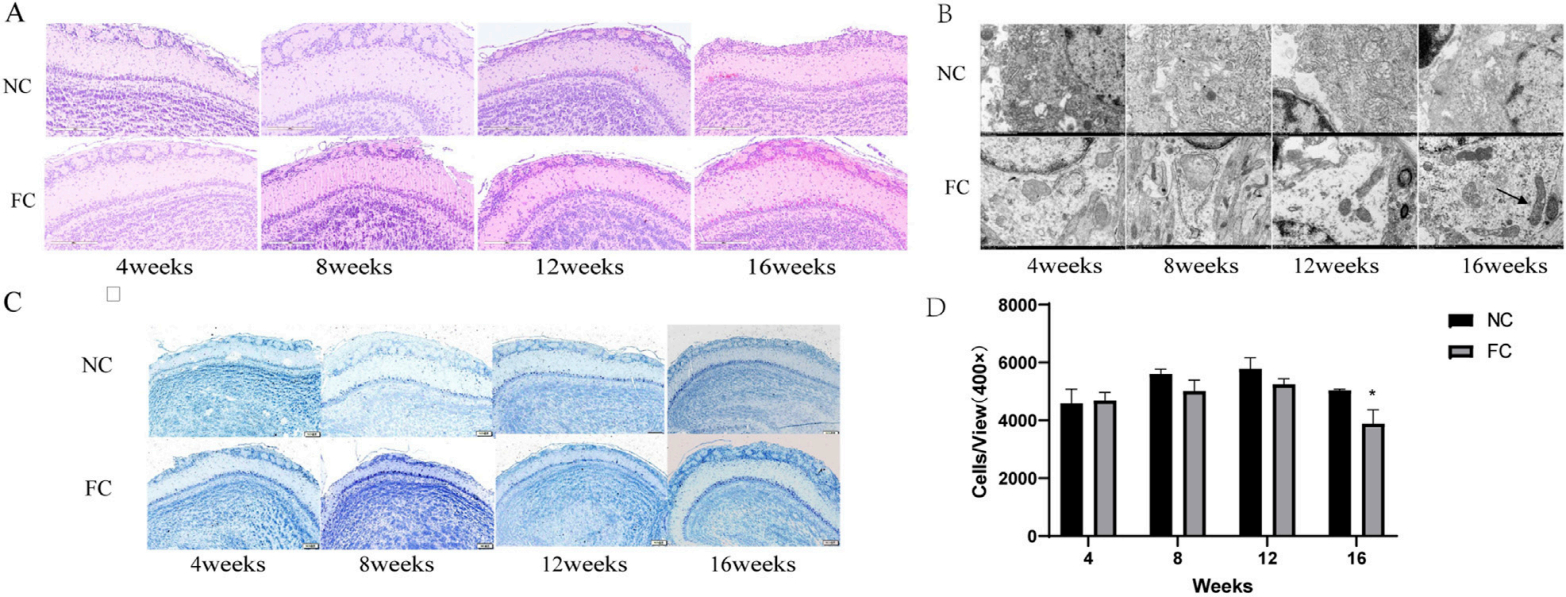
Impact of ferric citrate (FC) Treatment on neuronal integrity and mitochondrial morphology in the olfactory bulb over time. **(A)** Hematoxylin and eosin (HE) staining of olfactory bulb sections from NC (normal control) and FC-treated mice at 4, 8, 12, and 16 weeks. Up to 12 weeks, FC-treated mice exhibit normal histological architecture with an orderly cellular arrangement across all layers. By 16 weeks, there is a noticeable increase in eosinophilic neurons in the glomerular layer, indicating early signs of cellular pathology compared to the NC group. **(B)** Transmission electron microscopy images of the olfactory bulb at 4, 8, 12, and 16 weeks. At 4 weeks, mitochondria in FC-treated mice display normal morphology and electron density. By 8, 12, and 16 weeks, mitochondrial abnormalities are evident, including shrinkage, vacuolization (arrowheads), cristae loss (arrows), and increased electron density, contrasting with the NC group, which retains normal mitochondrial integrity. **(C)** Nissl staining of olfactory bulb sections showing neuronal morphology in the glomerular, mitral, and granule cell layers in both NC and FC-treated mice across all time points. Neuronal morphology remains largely unchanged up to 12 weeks, but at 16 weeks, the FC group shows altered neuronal staining patterns. **(D)** Quantitative analysis of neuron density in the olfactory bulb at 4, 8, 12, and 16 weeks. There is a significant reduction in neuron numbers in the FC-treated mice at 16 weeks compared to NC controls (*P < 0.05), suggesting progressive neuronal loss associated with prolonged iron overload. Neuron counts were determined per unit area (cells/mm^2^) at 400 × magnification.

Our study investigates the specific effects of chronic iron overload on the olfactory bulb, a brain region implicated in early neurodegenerative changes. We found that the olfactory bulb initially activates compensatory mechanisms to counteract iron toxicity, maintaining a normal histological structure with orderly cellular arrangement up to 12 weeks in FC-treated mice (Figure 3A. However, by 16 weeks, significant pathological changes, including an increase in eosinophilic neurons in the glomerular layer and a reduction in neuronal density, become apparent, as shown by Nissl staining (Figure 3C). Quantitative analysis confirms a marked decrease in neuron numbers in the FC-treated group compared to controls at 16 weeks (*P < 0.05; Figure 3D), revealing a transition from compensation to degeneration over time. These findings suggest that chronic iron overload leads to progressive neurodegeneration in the olfactory bulb following an initial phase of adaptation (Onukwufor et al., 2022).

A novel aspect of our study is the identification of early and progressive mitochondrial abnormalities preceding structural degeneration in the olfactory bulb. While mitochondrial morphology remains normal at 4 weeks, distinct signs of damage, such as shrinkage, vacuolization, and cristae loss, appear by 8 weeks and worsen significantly by 12 and 16 weeks (Figure 3B). These alterations indicate a disruption of mitochondrial function, likely driven by iron-induced ROS production, which compromises cellular energy metabolism and structural integrity (Hinarejos et al., 2020). The early onset of mitochondrial dysfunction suggests it plays a critical role in triggering the later neuronal damage observed in the olfactory bulb, emphasizing the importance of targeting iron-induced oxidative stress in early neurodegenerative interventions.

### 2.4 Selective colocalization of 4-HNE in the Granular and Glomeruli layers highlights neuronal vulnerability to oxidative damage in the olfactory bulb under chronic iron overload

**FIGURE 4:**
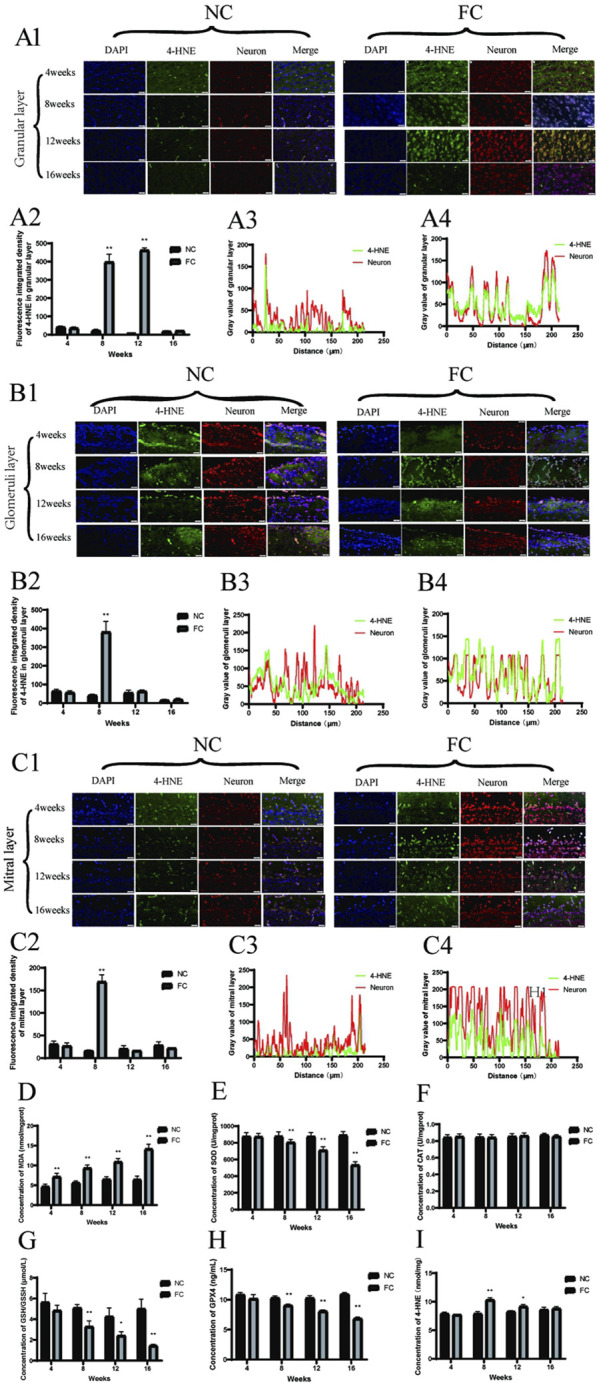
Oxidative stress and lipid peroxidation in the olfactory bulb of FC-treated mice. **(A)** 4-HNE and neuron colocalization in the granular Layer. Panel **(A1)** displays immunofluorescence images showing the colocalization of 4-HNE (green), a marker of lipid peroxidation, with neurons (red) in the granular layer of the olfactory bulb at 4, 8, 12, and 16 weeks in NC (control) and FC (ferric citrate-treated) mice. Panel **(A2)** presents the fluorescence intensity of 4-HNE in the granular layer, which is significantly higher in the FC group compared to the NC group at 8 and 12 weeks (**P < 0.01), indicating increased oxidative stress. Panels **(A3, A4)** illustrate the fluorescence intensity profiles of 4-HNE and neurons in the granular layer for the NC and FC groups, respectively, at 12 weeks, demonstrating stronger colocalization of 4-HNE with neurons in the FC group, suggesting more pronounced oxidative damage. **(B)** 4-HNE and neuron colocalization in the glomerular layer. Panel **(B1)** shows immunofluorescence images illustrating the colocalization of 4-HNE (green), a marker of lipid peroxidation, with neurons (red) in the glomerular layer of the olfactory bulb at 4, 8, 12, and 16 weeks in NC (control) and FC (ferric citrate-treated) mice. The FC group demonstrates a marked increase in colocalization beginning at 8 weeks, which is sustained through 12 weeks, indicating significant oxidative damage in this layer. Panel **(B2)** presents the quantitative analysis of 4-HNE fluorescence intensity in the glomerular layer, revealing significantly higher levels in the FC group at 8 weeks compared to the NC group (**P < 0.01), suggesting elevated oxidative stress. Panels B3 and B4 show the fluorescence intensity distribution profiles for 4-HNE and neurons in the glomerular layer for the NC and FC groups, respectively, at 8 weeks. **(B3)** demonstrates minimal colocalization in the NC group, whereas **(B4)** reveals a stronger overlap of 4-HNE and neuronal signals in the FC group at 8 weeks, reflecting increased lipid peroxidation and oxidative stress in the neurons. **(C)** 4-HNE and neuron colocalization in the mitral layer. Panel **(C1)** presents immunofluorescence images showing the colocalization of 4-HNE (green), a marker of lipid peroxidation, with neurons (red) in the mitral layer of the olfactory bulb at 4, 8, 12, and 16 weeks in NC (control) and FC (ferric citrate-treated) mice. While the FC group shows the highest 4-HNE fluorescence intensity at 8 weeks, indicating a peak in oxidative stress, the colocalization with neurons remains relatively weak, suggesting that lipid peroxidation is occurring but may not be confined to neuronal cells. Panel **(C2)** provides quantitative analysis of 4-HNE fluorescence intensity in the mitral layer, demonstrating a significant increase in the FC group at 8 weeks compared to the NC group (**P < 0.01), which continues to be elevated at 12 weeks, consistent with intensified oxidative stress. Panels **(C3, C4)** illustrate the fluorescence intensity distribution profiles for 4-HNE and neurons in the mitral layer at 8 weeks. **(C3)** shows minimal colocalization in the NC group, while **(C4)** indicates that, despite the high 4-HNE fluorescence intensity in the FC group, the overlap with neuronal signals is not strong, suggesting that the oxidative stress may also involve non-neuronal cells or extracellular components in this layer. **(D)** The concentration of MDA (malondialdehyde) in olfactory bulb, a marker of lipid peroxidation, shows a significant increase in the FC group compared to the NC group starting from week 4 and remains significantly elevated through week 16 (**P < 0.01), indicating persistent oxidative stress. **(E)** The activity of SOD (superoxide dismutase) in olfactory bulb is significantly reduced in the FC group beginning at week 8 and continuing through week 16 (**P < 0.01), reflecting compromised antioxidant defense against superoxide radicals. **(F)** The concentration of CAT (catalase) remains unchanged across all time points in both the FC and NC groups, indicating no significant impact on hydrogen peroxide detoxification capacity. **(G)** The concentration of GSH (glutathione) in olfactory bulb is significantly reduced in the FC group compared to the NC group, showing a marked decrease at week 8 and week 16 (**P < 0.01) and a moderate decrease at week 12 (*P < 0.05), suggesting depleted antioxidant reserves over time. **(H)** The activity of GPX4 (glutathione peroxidase 4) in olfactory bulb is significantly decreased in the FC group from week 8 onwards and remains lower through week 16 (**P < 0.01), indicating reduced enzymatic defense against oxidative stress. **(I)** ELISA analysis of 4-HNE levels in olfactory bulb, a biomarker of lipid peroxidation, demonstrates a significant increase in the FC group at week 8 (**P < 0.01) and remains elevated at week 12 (*P < 0.05), confirming heightened lipid peroxidation compared to the NC group.

The sustained elevation of oxidative stress markers and lipid peroxidation products in the olfactory bulb under chronic iron overload indicates cumulative and progressive oxidative damage. Our findings show that FC treatment significantly disrupts redox homeostasis, with a marked decline in antioxidant defenses such as the GSH/GSSH ratio starting at week 8 and continuing through weeks 12 and 16 (Figure 4G). This decline suggests that the olfactory bulb’s antioxidant capacity is increasingly compromised over time, likely due to the depletion of glutathione, a key intracellular antioxidant that protects against oxidative stress (Melo et al., 2011). However, there is no obvious impact on CAT, significant reduction in the activities of crucial antioxidant enzymes, such as superoxide dismutase (SOD) and glutathione peroxidase (GPX), from week 8 onwards (Figures 4E, F, H), reflecting a diminished capacity to neutralize reactive oxygen species (ROS) generated by iron overload. Moreover, malondialdehyde (MDA) levels, a lipid peroxidation byproduct, are elevated from week 4 and continue to increase through weeks 8, 12, and 16 (Figure 4D), aligning with the notion that iron catalyzes Fenton reactions, generating hydroxyl radicals that inflict oxidative damage on lipids, proteins, and DNA (Jansen Van Rensburg et al., 2021; Levi et al., 2024). The consistent rise in 4-hydroxynonenal (4-HNE) levels, another lipid peroxidation marker, from week 8 onwards (Figures 4D, I) further supports the hypothesis that antioxidant defenses are insufficient to counterbalance ongoing oxidative stress, thereby exacerbating neuronal vulnerability (Jurcau, 2021; Teleanu et al., 2022).

A deeper examination of colocalization patterns reveals that 4-HNE, a key marker of lipid peroxidation, is particularly concentrated in the Granular and Glomeruli layers of the olfactory bulb. Immunofluorescence analysis shows a pronounced increase in 4-HNE colocalization with neuronal markers in these layers from week 8 onwards, suggesting a region-specific vulnerability to oxidative damage (Figures 4A1–A4, B1–B4). This enhanced colocalization indicates that neurons in the Granular and Glomeruli layers are primary targets of iron-induced lipid peroxidation, leading to oxidative damage that disrupts neuronal integrity and function. In contrast, the Mitral layer exhibits high 4-HNE fluorescence intensity at 8 weeks (Figure 4C2), but with relatively weak colocalization with neurons (Figures 4C3, C4), implying that oxidative damage in this layer may also affect non-neuronal cells or extracellular components.

The selective concentration of 4-HNE in the Granular and Glomeruli layers underscores the distinct susceptibility of these neuronal populations to lipid peroxidation, likely due to their higher metabolic activity and iron uptake. This pattern of targeted oxidative damage is likely to impair membrane integrity, disrupt synaptic signaling, and initiate apoptotic pathways, thereby contributing to neuronal loss (Li et al., 2019; Nakamura et al., 2019). The differential colocalization profiles (Figures 4A3, B3, C3) further support the hypothesis that iron accumulation drives oxidative damage preferentially in regions with high metabolic demand, such as the olfactory bulb, where neuronal populations are more metabolically active and, therefore, more vulnerable to iron-induced oxidative stress (Cioffi et al., 2019). Our study provides new insights into the mechanisms underlying selective neuronal vulnerability in neurodegenerative diseases, highlighting that iron overload can induce targeted lipid peroxidation events, specifically affecting neuronal populations in certain olfactory bulb layers. The preferential accumulation of 4-HNE in the Granular and Glomeruli layers suggests that these regions may serve as early sites of neurodegenerative processes in diseases like Parkinson’s and Alzheimer’s, where oxidative stress plays a pivotal role (Subramaniam, 2019; Trist et al., 2019).

### 2.5 Caspase-3-mediated neuronal apoptosis in the granular layer of the olfactory bulb induced by iron overload

**FIGURE 5:**
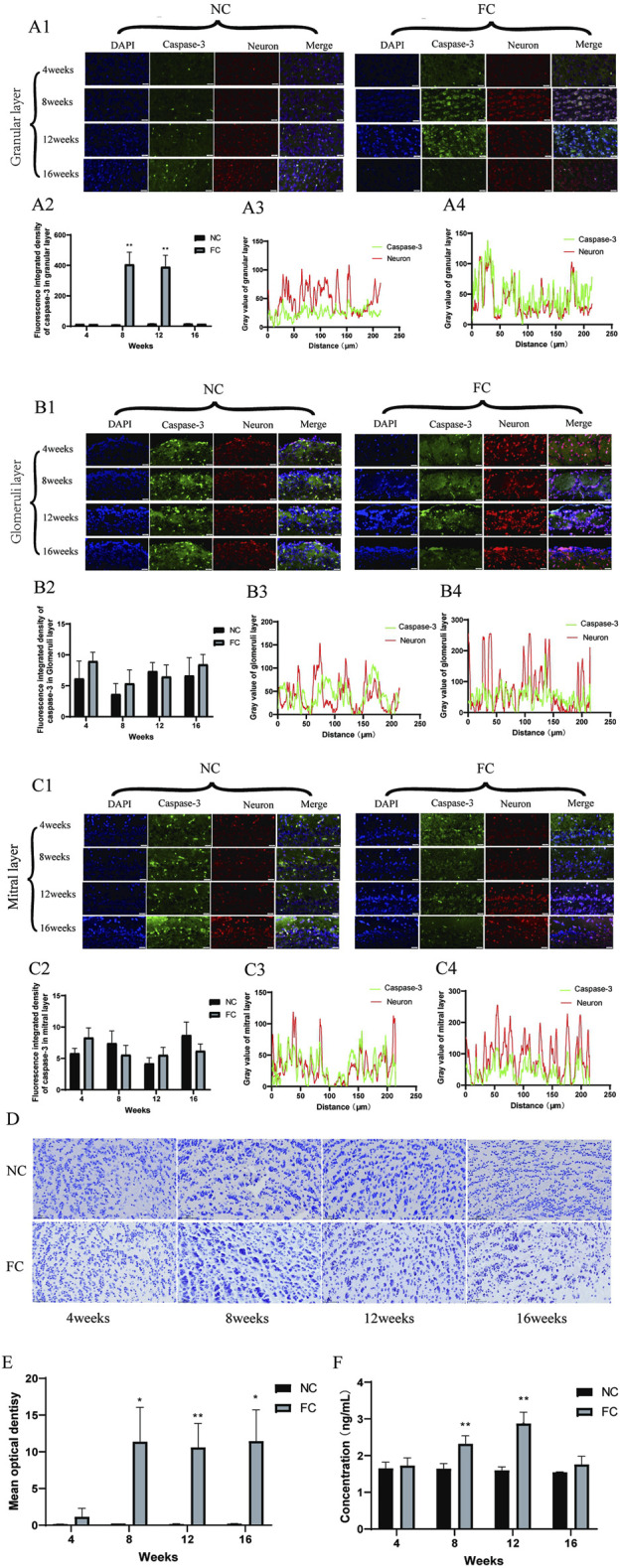
Iron Overload Induces Caspase-3-Mediated Neuronal Apoptosis in the Olfactory Bulb Verified by Immunofluorescence and TUNEL Analysis **(A)** Colocalization of Caspase-3 and Neurons in the Granular Layer. **(A1)** shows immunofluorescence images illustrating the colocalization of active caspase-3 (green), an apoptosis marker, with neurons (red) in the granular layer of the olfactory bulb in NC (control) and FC (ferric citrate-treated) mice at 4, 8, 12, and 16 weeks. **(A2)** quantifies caspase-3 fluorescence intensity, revealing significantly increased apoptosis in the FC group at 8 and 12 weeks (**p < 0.01). **(A3, A4)** display fluorescence intensity profiles at 8 weeks, showing enhanced colocalization of caspase-3 with neurons in the FC group, indicating elevated neuronal apoptosis due to iron overload. **(B)** Colocalization of Caspase-3 and Neurons in the Glomerular Layer. **(B1)** presents immunofluorescence images of active caspase-3 (green) colocalized with neurons (red) in the glomerular layer at 4, 8, 12, and 16 weeks for NC and FC groups. **(B2)** shows quantification at 8 weeks, demonstrating no significant difference between groups, suggesting minimal changes in apoptotic activity in this layer. **(B3, B4)** depict fluorescence intensity distribution profiles at 8 weeks, both showing limited colocalization, indicating low apoptotic activity in neurons within this layer. **(C)** Colocalization of Caspase-3 and Neurons in the Mitral Layer. **(C1)** displays immunofluorescence images of caspase-3 (green) colocalized with neurons (red) in the mitral layer at 4, 8, 12, and 16 weeks. **(C2)** quantifies fluorescence intensity at 8 weeks, revealing no significant differences between the FC and NC groups, suggesting comparable apoptosis levels. **(C3, C4)** show fluorescence intensity profiles at 8 weeks; both groups demonstrate limited colocalization, indicating that neuronal apoptosis is not prominent in this layer. **(D, E)** TUNEL Staining in the Olfactory Bulb. **(D)** presents TUNEL staining of olfactory bulb tissue in NC and FC mice at 4, 8, 12, and 16 weeks, marking DNA fragmentation as an indicator of apoptosis. **(E)** quantifies mean optical density of TUNEL-positive cells, showing significantly higher apoptosis levels in the FC group at 8, 12, and 16 weeks (*P < 0.05, **P < 0.01), confirming increased apoptotic activity due to iron overload. **(F)** Quantification of Caspase-3 protein concentration in olfactory bulb tissue from NC (control) and FC (ferric citrate-treated) mice at 4, 8, 12, and 16 weeks using ELISA. The data show a significant increase in Caspase-3 protein concentration in the FC group at 8 and 12 weeks compared to the NC group (**P < 0.01). However, at 16 weeks, the difference between the FC and NC groups was not significant, suggesting that apoptotic activity may plateau over time despite iron overload.

Chronic iron overload significantly activates apoptotic pathways in the olfactory bulb, primarily mediated by caspase-3. Immunofluorescence analysis demonstrates a clear trend of increased caspase-3 activation in the iron overload-treated (FC) group compared to the control (NC) group, particularly in the granular layer at 8 and 12 weeks (Figure 5A1). Although visual data suggest increased caspase-3 activation, quantitative fluorescence intensity analysis (Figure 5A2) reveals that the changes at 8 and 12 weeks are not statistically significant. This discrepancy suggests that while caspase-3 activation is visually apparent in specific regions, the overall apoptotic signal may be more localized, particularly in areas where iron-induced oxidative stress is most pronounced (Dusek et al., 2022; Zhang et al., 2020). This is further supported by the enhanced colocalization of caspase-3 with neuronal markers in the granular layer at 8 weeks (Figures 5A3, A4), indicating that oxidative stress directly triggers apoptosis in this region. These findings align with previous studies linking iron accumulation and oxidative stress to increased neuronal apoptosis in brain regions such as the hippocampus and cortex, which are similarly vulnerable to iron dysregulation in neurodegenerative conditions like Alzheimer’s disease (Ward et al., 2022; Zhang et al., 2020).

In contrast, the glomerular and mitral layers exhibit minimal colocalization of caspase-3 with neuronal markers (Figures 5B3, B4, C3, C4), indicating that apoptosis is primarily localized to the granular layer. This selective vulnerability may stem from localized differences in metabolic activity, iron handling, and oxidative stress responses, as suggested by previous research on neurodegenerative diseases. The ELISA data (Figure 5F) corroborate the immunofluorescence findings, showing a significant increase in caspase-3 levels at 8 and 12 weeks (**P < 0.01). However, by 16 weeks, caspase-3 levels decline, with no significant difference between the FC and NC groups, implying a potential reduction in apoptotic activity. This decline could result from the depletion of neurons susceptible to apoptosis or from compensatory mechanisms, such as the upregulation of cellular defenses like antioxidant pathways, as observed in chronic oxidative stress models. These findings suggest that apoptosis may peak during the initial phase of iron overload but later stabilize as neurons either adapt to the oxidative environment or are lost due to apoptosis.

TUNEL staining further supports these conclusions, revealing increased DNA fragmentation—a hallmark of apoptosis—from week 8 onwards in the FC group (Figure 5D). Quantification of TUNEL-positive cells (Figure 5E) demonstrates a significant rise in apoptotic cells at 8 and 12 weeks, particularly in the granular layer (*P < 0.05, **P < 0.01). However, by 16 weeks, both caspase-3 levels (Figure 5F) and TUNEL-positive cells decrease, suggesting that apoptotic activity declines as the neuronal population is reduced or becomes more resistant to further damage. This pattern of apoptotic activity suggests a peak at 12 weeks, followed by a reduction in apoptosis as the remaining neurons adapt to the persistent iron-induced oxidative stress. These findings are consistent with other studies demonstrating that prolonged iron overload initially triggers robust apoptotic responses, but over time, compensatory pathways mitigate further cell death through mechanisms such as enhanced antioxidant defenses.

## 3 Discussion

### 3.1 Selective iron accumulation and its neurodegenerative implications

Our study highlights the olfactory bulb’s unique susceptibility to chronic iron overload, with progressive iron accumulation beginning at week 8 and peaking at week 16 (Figures 1A, B, E). This finding is consistent with prior studies that demonstrate how regions with high metabolic rates and dopaminergic activity are particularly prone to oxidative damage due to iron dysregulation (Carocci et al., 2018; Checa and Aran, 2020; Jiang et al., 2017). High metabolic rates demand an increase in oxygen consumption (Akhigbe and Ajayi, 2021), which produces reactive oxygen species (ROS). When combined with dysregulated iron handling, this leads to oxidative stress, which further exacerbates neurodegeneration through mechanisms such as lipid peroxidation, protein modification, and DNA damage (David et al., 2022; Hinarejos et al., 2020). Iron catalyzes the Fenton reaction, converting hydrogen peroxide into highly reactive hydroxyl radicals, thereby amplifying oxidative damage in vulnerable brain regions (Song et al., 2020). Previous studies on Parkinson’s disease have shown that regions like the substantia nigra are particularly vulnerable to iron-induced oxidative damage due to their high dopaminergic activity (Ma et al., 2021; Wise et al., 2022), which mirrors the situation we observe in the olfactory bulb. Dopaminergic neurons are known to accumulate neuromelanin, which binds iron, and under conditions of iron overload, this interaction becomes pathological, leading to increased oxidative stress and cell death (Moreno-García et al., 2021). Our findings suggest that the olfactory bulb, which shares similar dopaminergic activity (Capsoni et al., 2021; Korshunov et al., 2020), may exhibit parallel vulnerabilities.

The selective accumulation of iron in the olfactory bulb compared to peripheral organs such as the liver and spleen (Figure 1B) further supports the idea of regional variation in iron metabolism. This regional specificity is well documented in neurodegenerative disease research, where various brain regions exhibit different susceptibilities to iron dysregulation. For instance, the literature described how regions like the hippocampus and cortex handle iron differently due to variations in iron transporter and storage protein expression (Youdim, 2008). These findings underscore that not all brain regions are equally equipped to manage iron homeostasis, explaining why some areas, like the olfactory bulb and substantia nigra, are particularly prone to neurodegeneration in the context of iron overload (Yuan et al., 2023).

### 3.2 Disrupted iron homeostasis and compensatory mechanisms

Iron overload profoundly disrupts iron homeostasis in the olfactory bulb, as evidenced by the upregulation of key iron-handling proteins, including ferritin light chain (FTL), transferrin receptor (TFRC), and ferroportin (FPN) (Figures 2A–C). This upregulation is consistent with the brain’s adaptive response to elevated iron levels, as described in studies on neurodegeneration, where increased expression of ferritin helps sequester free iron, thereby reducing its availability for catalyzing harmful oxidative reactions (Arosio et al., 2009; Jomova and Valko, 2011). However, while this initial response serves a protective role, the continued elevation of these proteins by week 16 suggests that the system’s compensatory capacity has been overwhelmed. Excessive iron storage leads to intracellular stress, as ferritin and other iron regulators reach their limit, unable to adequately buffer the increasing iron load. This shift from adaptive to maladaptive iron regulation parallels observations in diseases like Alzheimer’s and Parkinson’s, where disrupted iron metabolism exacerbates oxidative stress and contributes to neurodegeneration (Belaidi and Bush, 2016; Jiang et al., 2017).

The failure of these compensatory mechanisms is further underscored by the upregulation of oxidative stress markers such as SLC7A11 and ACSL4 (Figures 2J, K), both of which are heavily involved in the cellular response to oxidative damage. SLC7A11 is part of the system responsible for maintaining glutathione levels, a crucial antioxidant, while ACSL4 is implicated in the synthesis of polyunsaturated fatty acids that are prone to lipid peroxidation. The elevated expression of these markers highlights the inability of endogenous defenses to counteract the damaging effects of iron-induced oxidative stress. Dixon et al. have shown that lipid peroxidation products, such as 4-HNE, accumulate under conditions of excessive iron, further disrupting cellular structures and exacerbating neuronal death through ferroptosis (David et al., 2022; Zhang et al., 2020). The persistent oxidative stress seen in our study reflects a critical tipping point at which the olfactory bulb’s defenses are not just insufficient but actively contribute to the pathological cycle of damage, a phenomenon similarly observed in various neurodegenerative disorders (Perluigi et al., 2024; Li et al., 2013). These findings have significant implications for therapeutic strategies targeting iron-induced neurodegeneration. While iron chelation therapies have been explored to reduce iron accumulation, our results suggest that a multifaceted approach is necessary. Therapies aimed at not only regulating iron homeostasis but also enhancing antioxidant defenses and inhibiting ferroptosis could provide more comprehensive protection against progressive neuronal damage. Rochette et al. discuss the potential benefits of combining iron chelators with ferroptosis inhibitors, such as ferrostatin-1, to mitigate the effects of lipid peroxidation and oxidative stress (Rochette et al., 2022). Thus, addressing both the cause (iron dysregulation) and the consequence (oxidative damage) of iron overload could offer a more effective approach to treating neurodegenerative diseases where iron toxicity plays a pivotal role.

### 3.3 Lipid peroxidation as a driver of neuronal apoptosis and ferroptosis

Our study uncovers the significant contribution of lipid peroxidation to both apoptotic and ferroptotic pathways in iron-induced neurodegeneration within the olfactory bulb, shedding light on how oxidative damage acts as a central driver of neuronal death. Elevated levels of malondialdehyde (MDA) and 4-hydroxynonenal (4-HNE), key biomarkers of lipid peroxidation, reflect persistent oxidative damage to neuronal membranes, particularly in the granular and glomerular layers (Figure 4D). These findings align with existing research that underscores the role of lipid peroxidation products like 4-HNE in initiating apoptosis by disrupting membrane integrity and triggering caspase activation (Łuczaj et al., 2017; Schaur et al., 2015; Wójcik et al., 2020). The localization of 4-HNE to neurons in these layers (Figures 4A1–A4, B1–B4) suggests that the neurons in metabolically active regions with high iron content are more susceptible to iron-induced oxidative damage. Studies have shown that iron accumulates in regions with intense synaptic activity and high metabolic rates, making these neurons particularly vulnerable to oxidative stress (Ceylan et al., 2019; Garza-Lombó et al., 2018; Yarjanli et al., 2017). This susceptibility is exacerbated by the role of iron in catalyzing the Fenton reaction, which amplifies ROS production and accelerates lipid peroxidation, further compromising neuronal survival (Kajarabille and Latunde-Dada, 2019; Villalón-García et al., 2023).

The initial upregulation of caspase-3 in the granular layer at week 8 (Figures 5A1–A4) underscores the role of apoptosis as an early response to iron-induced oxidative stress. Mattson and colleagues have shown that oxidative stress activates caspase-3, a central executioner of apoptosis, by triggering mitochondrial dysfunction and cytochrome c release, leading to programmed neuronal death (Bhatia and Sharma, 2021; Dionísio et al., 2021). This early peak in apoptotic activity aligns with our observation that, by week 16, caspase-3 levels decline (Figure 5F), suggesting that apoptosis is an acute response to initial iron overload but diminishes as vulnerable neurons are eliminated or as protective compensatory mechanisms are activated. However, it is crucial to note that apoptosis alone does not account for the full extent of neuronal death observed in the olfactory bulb. The persistence of oxidative damage, as indicated by sustained 4-HNE and ACSL4 elevation, points to ferroptosis—an iron-dependent form of cell death driven by lipid peroxidation—as a critical factor in ongoing neurodegeneration (Costa et al., 2023a; Costa et al., 2023b; Hassannia et al., 2021).

Ferroptosis plays a distinct role in neurodegeneration, particularly when apoptosis subsides, highlighting the multifaceted nature of iron-induced cell death. While apoptosis is regulated by caspase pathways and typically triggered by mitochondrial dysfunction, ferroptosis is characterized by the accumulation of lipid peroxides and the failure of cellular antioxidant defenses, such as glutathione (GSH) depletion and GPX4 inactivation (Kajarabille and Latunde-Dada, 2019; Su et al., 2019). The upregulation of ACSL4, a key enzyme involved in the synthesis of polyunsaturated fatty acids (PUFAs), which are highly susceptible to peroxidation, emphasizes the role of lipid metabolism in sustaining ferroptosis in the olfactory bulb (Figures 2J, K). Ajoolabady et al. (2023) demonstrated that ACSL4 promotes the incorporation of PUFAs into membrane phospholipids, making them prime targets for lipid peroxidation and ferroptosis under conditions of iron overload and oxidative stress. In our study, the persistence of mitochondrial abnormalities, along with elevated lipid peroxidation markers from week 8 onwards, supports the idea that ferroptosis, rather than apoptosis, becomes the dominant mode of cell death as the neurodegenerative process progresses (Figure 3B).

The unique vulnerability of the olfactory bulb to iron-induced oxidative damage, with apoptosis peaking early and ferroptosis sustaining long-term neuronal loss, emphasizes the complexity of the cell death mechanisms involved. This interplay between apoptosis and ferroptosis, both driven by lipid peroxidation, underscores the importance of addressing both pathways in potential therapeutic interventions. Iron chelation alone may not be sufficient; targeting the lipid peroxidation pathway, particularly through the inhibition of ACSL4 and the use of ferroptosis inhibitors like ferrostatin-1, could offer a more comprehensive neuroprotective strategy (Han et al., 2020; Lv et al., 2023). Furthermore, our study shows that oxidative damage in the mitral layer, where 4-HNE fluorescence is high but neuronal colocalization is weak (Figures 4C1–C4), suggests that non-neuronal cells, such as glial cells, also play a role in propagating neurodegeneration. Leszek et al. (2016) explored how glial cells, especially microglia and astrocytes, respond to oxidative stress by releasing pro-inflammatory cytokines that exacerbate neuronal injury, contributing to a cycle of inflammation and oxidative damage in neurodegenerative diseases. This insight into the involvement of non-neuronal cells adds another layer to the understanding of how iron-induced oxidative stress drives both neuronal and non-neuronal cell death, making it essential for future therapeutic approaches to also consider the role of glial cells in the pathogenesis of iron-related neurodegeneration.

### 3.4 Implications for therapeutic strategies and future research

The selective vulnerability of the olfactory bulb to iron-induced oxidative damage has significant implications for developing targeted therapeutic strategies. Our findings suggest that therapies aimed at modulating iron levels, enhancing antioxidant defenses, and inhibiting both apoptosis and ferroptosis could be critical in mitigating neurodegeneration in conditions like Alzheimer’s and Parkinson’s diseases. Our study provides compelling evidence that chronic iron overload in the olfactory bulb drives neurodegeneration through a combination of disrupted iron homeostasis, oxidative stress, lipid peroxidation, and the activation of both apoptotic and ferroptotic pathways. Future research should focus on elucidating the molecular mechanisms that govern iron transport and cell death regulation across different brain regions. Such studies could pave the way for early diagnostic tools and more effective neuroprotective interventions in neurodegenerative diseases.

## 4 Conclusion

Our study employed a comprehensive set of advanced imaging and biochemical techniques to investigate the impact of chronic iron overload on the olfactory bulb in middle-aged mice. Through the use of inductively coupled plasma mass spectrometry (ICP-MS) and Perls’ Prussian blue staining, we accurately quantified and localized iron accumulation over a 16-week period. This analysis revealed a progressive buildup of iron within the olfactory bulb, which peaked at week 16. Parallel assessments of oxidative stress markers, including 4-hydroxynonenal (4-HNE) and malondialdehyde (MDA), indicated significant lipid peroxidation and neuronal damage over time. Additionally, transmission electron microscopy (TEM) identified early and progressive mitochondrial abnormalities, signaling disrupted cellular energy metabolism. These structural and biochemical changes were further associated with impaired olfactory function, as evidenced by the food-burial test, which showed a decline in sensory performance from week 12 onwards. Moreover, we observed the upregulation of key iron metabolism proteins (FTL, TFRC, FPN) and the activation of apoptotic pathways, particularly caspase-3, confirming the initiation of iron-induced neuronal apoptosis. Collectively, these findings illustrate the heightened vulnerability of the olfactory bulb to iron overload, providing crucial insights into the early mechanisms of neurodegeneration and underscoring the potential role of the olfactory bulb as an early biomarker for neurodegenerative diseases.

## 5 Materials and methods

### 5.1 Animals

All animal works were performed in accordance with the requirements of “The National Institutes of Health Guide for the Care and Use of Laboratory Animal” and all procedures were approved by the Institutional Animal Care and Use Committee of Sichuan Institute for Drug Control, China [Approval No. (IACUC-2020-KYYL-013)]. The mice were obtained from Beijing Vital River Laboratory Animal Technology Co., Ltd. (SCXK (京) 2016-0011) and maintained in individual cages in a specific pathogen-free environment with an automatically controlled 12-hour light/dark cycle and free access to food and water for 7 days. 96 female C57BL/6 mice were randomly divided into a control group (n = 48) and a model group (n = 48). The model group received daily oral administration of the test compound for 16 weeks, while the control group received an equivalent volume of distilled water. Every 4 weeks, 12 mice from each group were selected for behavioral testing, followed by euthanasia for further analysis. Mice were anesthetized with Zoletil (50 mg/kg, intraperitoneally) to ensure they were fully unconscious before euthanasia by cervical dislocation.

### 5.2 Detection of iron

A tissue sample weighing 0.05 g was added to 1 mL of concentrated nitric acid and 0.5 mL of hydrogen peroxide in a capped vessel. The mixture was transferred to a microwave digestion vessel containing 8 mL of deionized water and subjected to microwave digestion (CLASSIC, MARS6) for 100 min. Upon completion of digestion, the solution was diluted with deionized water to a final volume of 2 mL. A calibration curve was constructed using standard solutions at concentrations of 6.4 μg/mL, 3.2 μg/mL, 1.6 μg/mL, 0.8 μg/mL, 0.4 μg/mL, 0.2 μg/mL, 0.1 μg/mL, and 0.05 μg/mL. Subsequently, 500 μL of each sample—blank control, iron standard 1, iron standard 2, calibration standards (stock solution), and the test samples—was combined with 4.5 mL of diluent to achieve a final volume of 5 mL. These prepared samples were then sequentially loaded into the sample rack of the inductively coupled plasma mass spectrometer (ICP-MS; Agilent Technologies, 1262–6420) for analysis.

### 5.3 Olfactory discrimination test

The olfactory discrimination ability of the rats was evaluated using an olfactory discrimination task adapted from a previously described protocol (Zhang et al., 2022). In this task, each rat was placed for 5 min in a cage divided into two identical compartments (30 cm × 30 cm × 20 cm) separated by an open door. One compartment contained fresh sawdust (novel), while the other contained sawdust (familiar) that the same rat had occupied for 48 h prior to the test. The time (in seconds) spent by the rat in each compartment (familiar vs. novel) was recorded to assess its olfactory discrimination performance.

### 5.4 Food burying test

The assessment of olfactory function in the context of autoimmune central nervous system neuroinflammation was conducted using a food burying test, which measures the time required to locate a buried food pellet, as previously described (Odland et al., 2023). Briefly, mice were placed in a clean cage (42 cm × 28 cm × 18.5 cm) and exposed to the food pellet for 2–3 consecutive days before testing. Mice were fasted for 18–24 h prior to the test and acclimated to the testing room for 1 h. The test commenced by placing a subject mouse in a clean cage filled with 3 cm of fresh bedding. After a 1-hour habituation period, a food pellet was buried 0.5 cm beneath the bedding. The mouse was then removed from its home cage, positioned at the center of the test cage, and the timer was started. The latency to locate the pellet was recorded. If a mouse failed to find the pellet within 5 min, the trial was terminated, and a maximum time of 300 s was assigned. The latency time for each mouse was measured both before immunization (1 day prior) and at day 33 post-immunization (recovery stage).

### 5.5 Hematoxylin and eosin (H&E) staining

The olfactory bulb tissue was fixed in 4% paraformaldehyde (PFA) and subsequently dehydrated through a graded ethanol series. The tissue was then embedded in paraffin and sectioned into 5 μm-thick slices. Following deparaffinization to water, the sections were stained with hematoxylin and eosin, dehydrated through a graded alcohol series, cleared with xylene, and mounted using neutral balsam. The stained sections were scanned using a Leica digital pathology system (Leica, Aperio GT450).

### 5.6 Nissl staining

Paraffin-embedded sections were deparaffinized to water and stained following the protocol provided by the Nissl staining kit (Methylene Blue Method; Solarbio, G1434). After staining, the sections were dehydrated in a graded series of alcohols, cleared in xylene, and mounted with neutral balsam. The sections were scanned using the Leica digital pathology system (Leica, Aperio GT450). Neuronal counts were performed using ImageJ (Win-64) on 10 randomly selected high-power fields per section.

### 5.7 Prussian blue staining

Paraffin sections were deparaffinized to water and stained according to the manufacturer’s instructions using the Prussian Blue Iron Stain Kit (Ferric Iron, Enhanced with DAB; Solarbio, G1328). The sections were counterstained with hematoxylin, dehydrated through a graded series of ethanol, cleared with xylene, and mounted with neutral balsam. Scanning was performed with the Leica digital pathology system (Leica, Aperio GT450). The percentage of iron-positive cells was quantified using ImageJ (Win-64).

### 5.8 Transmission electron microscopy (TEM)

The olfactory bulb tissues were fixed in 2.5% glutaraldehyde, rinsed three times with phosphate-buffered saline (PBS), and post-fixed in 1% osmium tetroxide (OsO4) for 1 h. The samples were dehydrated through a graded ethanol series, embedded in Epon 812 resin, and sectioned into ultrathin slices. Sections were stained with 2% uranyl acetate and lead citrate and then examined under a transmission electron microscope (JEOL, JEM-1200EX). Images were captured to assess the ultrastructural morphology of the olfactory bulb.

### 5.9 TUNEL assay

Paraffin-embedded tissue sections were deparaffinized, rehydrated to water, and subjected to terminal deoxynucleotidyl transferase dUTP nick-end labeling (TUNEL) staining using a TUNEL Apoptosis Detection Kit (Beyotime, C1098) according to the manufacturer’s protocol. After TUNEL labeling, diaminobenzidine (DAB) substrate was applied for chromogenic detection. The stained sections were subsequently scanned using a G Cell Technology Scanner (G Cell Technology, GScan-60).

### 5.10 Immunohistochemistry (IHC)

Paraffin sections were deparaffinized, rehydrated, and immersed in 3% hydrogen peroxide (H₂O₂) to block endogenous peroxidase activity. Following rinsing in distilled water, antigen retrieval was performed in a sodium citrate buffer under high-pressure conditions. After a second wash in distilled water, the sections were blocked with bovine serum albumin (BSA) for 40 min, using the Mouse/Rabbit IgG Immunohistochemistry Kit (SABC, ready-to-use; BOSTER, SA1020). Primary antibodies were applied against ferritin light chain (FTL, Genetex, GTX112943), ferroportin (SLC40A1, Proteintech, 26601-1-AP), and transferrin receptor (CD71, Proteintech, 10084-2-AP) and incubated overnight at 4°C. The sections were then washed in PBS and incubated with HRP-conjugated secondary antibodies from the same kit for 40 min, followed by additional PBS washes and a 40-min incubation with the SABC complex. DAB substrate was applied for color development. After dehydration through a graded ethanol series, the sections were cleared, mounted, and scanned with a G Cell Technology Scanner. Quantification of positive staining was performed by measuring integrated optical density using ImageJ (Win-64).

### 5.11 Immunofluorescence co-localization

Paraffin-embedded sections were deparaffinized, rehydrated, and subjected to antigen retrieval using sodium citrate buffer under high-pressure conditions. Sections were rinsed in distilled water and blocked with BSA for 40 min. The sections were then incubated with primary antibodies against 4-hydroxynonenal (4-HNE, mouse monoclonal; Abcam, ab48506) and cleaved caspase-3 (rabbit polyclonal; Santa Cruz, sc-7272) overnight at 4°C. After PBS washes, the sections were incubated with Alexa Fluor 488-conjugated donkey anti-rabbit IgG secondary antibody (Invitrogen, A21206) for 40 min. Following additional PBS washes, sections were re-blocked with BSA for 40 min, then incubated with a primary antibody against neuron-specific marker (rabbit monoclonal; Abcam, ab177487) overnight at 4°C. After further PBS washes, the sections were incubated with Alexa Fluor 594-conjugated goat anti-rabbit IgG secondary antibody (Invitrogen, A11037) for 40 min. The sections were washed with PBS, mounted with an anti-fade mounting medium containing DAPI, and imaged using an OLYMPUS VS200 digital slide scanner.

### 5.12 Measurement of biochemical indicators

Levels of malondialdehyde (MDA; NJJCBIO, A003-1-2), superoxide dismutase (SOD; NJJCBIO, A001-3-2), glutathione (GSH/GSSG; NJJCBIO, A061-1-2) and tissue iron (Tissue Iron Assay Kit; NJJCBIO, A039-2-1) were determined following the protocols provided by the respective assay kits. Absorbance measurements were performed using a Thermo Varioskan Flash spectrophotometer. The concentrations of glutathione peroxidase 4 (GPX-4; NJJCBIO, H545-1-2), caspase3 (JL10456-96T) and 4-hydroxynonenal (4-HNE) were specifically quantified using enzyme-linked immunosorbent assay (ELISA) kits.

### 5.13 Western blot

Tissue samples (∼20 mg) were homogenized in protein lysis buffer using an ultrasonic cell disruptor (SCIENTZ, 1200E). The homogenates were centrifuged at 10,000 × g for 10 min at 4°C to collect the supernatants, and protein concentrations were measured with a NanoOne microvolume spectrophotometer (Yooning). Equal amounts of protein were separated by SDS-PAGE (KeyGEN BioTECH, KGC4401-50) and transferred onto PVDF membranes. The membranes were blocked with 5% non-fat milk and incubated overnight at 4°C with primary antibodies specific for FTL, FPN, TFRC, 4-HNE, Caspase-3, and Caspase-9 (dilution 1:1,000). After washing, membranes were treated with an HRP-conjugated secondary antibody (1:5,000) for 1.5 h at room temperature. Proteins were detected using an ECL substrate (SuperKine, BMU102-CN) and visualized with the ChemiDoc MP Imaging System (Bio-Rad, B818). Densitometric analysis was performed using ImageJ software.

### 5.14 Statistical analysis

Data were presented as means ± standard deviations for eight samples. The normality of the variables was assessed using the Kolmogorov–Smirnov test, and homogeneity of variance was evaluated using the Levene’s test. As the data showed normal distribution and homogeneity of variance, they were analyzed using one-way analysis of variance (ANOVA), followed by Tukey’s *post hoc* tests for multiple comparisons. A *p*-value of less than 0.05 was regarded as statistically significant.

## Data Availability

The original contributions presented in the study are included in the article/supplementary material, further inquiries can be directed to the corresponding authors.
